# Mapping barriers, enablers and implementation determinants to shared models of care for physical health and sexual wellbeing among young people with mental health difficulties using the Consolidated Framework for Implementation Research: A scoping review protocol

**DOI:** 10.12688/hrbopenres.14032.2

**Published:** 2025-05-12

**Authors:** Allyson J Gallant, John Paul Lyne, Karen O'Connor, Greg Sheaf, Shaakya Anand-Vembar, Donal O'Keeffe, Caroline Wilson, Yulia Kartalova- O'Doherty, Louise Doyle, Mary Cannon, Leona Ryan, Gary Donohoe, David McEvoy, David Cotter, Olivia Longe, Colm McDonald, Agnes Higgins, Rebecca Murphy, Sara Burke, Catherine D Darker

**Affiliations:** 1 Public Health & Primary Care, The University of Dublin Trinity College, Dublin, Leinster, Ireland; 2 Department of Psychiatry, Royal College of Surgeons in Ireland 26 York Street Campus, Dublin, Leinster, Ireland; 3 University College Cork, Cork, County Cork, Ireland; 4 School of Nursing & Midwifery, The University of Dublin Trinity College, Dublin, Leinster, Ireland; 5 Mental Health Ireland, Dublin, Ireland; 6 ARCHES Recovery College, Dublin, Ireland; 7 School of Psychology, University of Galway, Galway, County Galway, Ireland; 8 School of Medicine, University of Galway, Galway, County Galway, Ireland; 9 Dublin City University School of Nursing Psychotherapy and Community Health, Dublin, Leinster, Ireland

**Keywords:** Adolescent, CFIR, delivery of healthcare, general health, implementation science, mental health, review, sexual health

## Abstract

**Background:**

Approximately one in eight people live with mental health difficulties, with onset commonly occurring in youth. It is critical to ensure care addresses all aspects of health, including physical health and sexual wellbeing needs, to achieve positive recovery outcomes. Connecting primary and secondary healthcare providers and service users through shared models of care is a critical aspect of this. The objectives of this scoping review will be to 1) identify and describe the implementation of shared models of care which address the mental health of young people and their physical health and/or sexual wellbeing needs, and 2) identify the determinants of implementing these models of care.

**Protocol:**

Following Joanna Briggs Institute guidelines, studies will be included if they describe shared models of care for young people (aged 10–25) in any healthcare setting, specifically addressing mental health and physical health or sexual wellbeing needs. The review will employ the Consolidated Framework for Implementation Research (CFIR) to organise and assess findings. A librarian developed the search strategy, which will be applied to Web of Science, Medline, Embase, CINAHL, and PsycINFO databases. Two independent reviewers will screen titles, abstracts and full texts, followed by data extraction and critical appraisal of included studies. Discrepancies at all stages will be resolved through discussion or by a third reviewer. Screening results will be summarised in a PRISMA flow diagram. Narrative summaries, supported by tables and figures where applicable, will address the review’s objectives. Findings will undergo thematic analysis, with implementation determinants mapped deductively to CFIR.

**Discussion:**

Findings will inform the adaptation of implementation strategies to support the implementation of policy for improving healthcare delivery to young people with mental health difficulties.

**Registration:**

Open Science Framework ( osf.io/rj783).

## Background

Mental health difficulties are prevalent in society, with the World Health Organisation (WHO) estimating one in eight people globally live with a mental health condition ^1, 2^. Mental health difficulties are dynamic and can occur across the life course; however, onset typically occurs by age 25, with over half of those experiencing symptoms before age 15 ^3, 4^. Recent studies have indicated an even higher prevalence of general mental health difficulties in young people; for example, data from Ireland in 2019 found 63% of young people aged 10–24 years experienced anxiety and depression, with 21% of those aged 10–14 years having experienced a mental health difficulty (e.g., anxiety, conduct disorders) ^5^. As adolescence and early adulthood are periods of transition, ensuring this cohort has access to timely, adequate and quality mental healthcare is essential to supporting short-term and long-term health outcomes. However, provision of mental healthcare is often fragmented, resulting in poor patient outcomes and ineffective health system use ^6– 8^. The COVID-19 pandemic had detrimental effects on mental health, with an estimated 25% global increase in anxiety and depression in 2020, and with 30% of young people across Europe reporting the pandemic had negative effects on their mental health and wellbeing ^9, 10^. As the prevalence of mental health difficulties is a growing global health concern, it is essential to support prevention, early intervention, and resiliency, particularly among young people ^11^.

As a young person adjusts to the symptoms of their mental health difficulties and/or diagnosis, attention is needed to their holistic health, including their physical health and sexual wellbeing. Physical health is the “ *condition of your body, taking into consideration everything from the absence of disease to fitness level*” ^12^. Physical health among those with mental health difficulties can be negatively affected by both individual lifestyle factors (e.g., smoking status, poor diet quality, sedentary behaviours) and treatment plans (e.g., medications) ^13^. For example, those with severe mental health difficulties (e.g., schizophrenia, bipolar disorder) are particularly vulnerable to several physical conditions as antipsychotic medications can contribute to the development of chronic illnesses such as premature cardiovascular disease ^14, 15^. Young people taking antipsychotics for at least six months are more likely to experience increases in weight, fasting glucose, cholesterols, and triglyceride levels compared to adults on the same medication ^16^, putting them at risk for chronic diseases, including obesity, cardiovascular disease and type 2 diabetes ^17, 18^. Despite the higher risk for these physical comorbidities, there is inadequate screening or monitoring for these conditions in young people across primary and secondary care ^19– 21^. Recent research highlighted young people with mental health difficulties often have limited knowledge of the effects their mental health treatments can have on their physical health, and felt inadequately informed by their healthcare providers ^14^. These unmet physical health needs may contribute to the “scandal of premature mortality,” ^22^ where those with mental health difficulties experience a reduced life expectancy by 15–20 years compared to the general population ^17, 23, 24^.

In addition to mental and physical health, the sexual wellbeing of young people with mental health difficulties should be addressed ^25^. As emerging mental health symptoms and diagnoses can coincide with the onset of puberty, sexual wellbeing should be considered a priority for this population. Sexual health and wellbeing are interrelated concepts in public health, with the focus sexual health on fertility management, prevention and management of sexually transmitted infection (STI) and prevention of sexualised violence, while sexual wellbeing encompasses key factors such as sexual self-esteem, comfort with sexuality and sexual safety ^26^. Common sexual health concerns among youth globally include access and use of contraception, unintended pregnancy and contracting or transmitting STIs ^27^. Young people with mental health difficulties are at an increased risk of engaging in these high-risk sexual behaviours compared to their peers ^28– 30^.

Collaboration is needed within and across the health system to improve overall health outcomes among young people experiencing mental health difficulties, with particular attention to their physical health needs and sexual wellbeing. A shared model of care is the collaboration between primary care (e.g., general practitioners) and secondary care (e.g., specialist mental health services) to support patient referrals, assessments and diagnoses, treatment and monitoring, and discharge planning ^31, 32^. This approach to care can exist across a continuum, from patient care coordination to full physical integration of services and shared electronic patient medical records ^31, 33^. Shared models of care have been associated with improvements in young people’s clinical and quality of life outcomes, and engagement with treatment from patients and their families ^34^. Additional organisational and health system level outcomes include cost-effective care and improved access to, and provision of, quality healthcare ^35, 36^. Therefore, many of the underlying factors associated with the “scandal of premature mortality” could be addressed using shared models of care. A consistent recommendation in Ireland’s national mental health policies for over a decade has been the use of shared models of care between primary and specialist secondary care to address individuals’ mental illness and accompanying health needs ^11, 32, 37^; however this model has yet to be implemented. With a strong evidence base to support the use of shared model of care and the positive effects on individual, organisational and system-level outcomes, it is worth investing in this approach to healthcare coordination and continuity of care. Yet translating this policy recommendation into practice has remained a challenge in the country.

Implementation science is a growing field of applied research to improve the uptake of evidence into practice ^38^, and offers a route to support the delivery of shared models to address the physical health and sexual wellbeing needs of young people with mental health difficulties. Central to implementation science is identifying barriers and enablers to adopting best practice interventions ^39– 41^, and developing tailored, context-specific strategies to address recognised barriers and leverage enablers to facilitate the adoption of new clinical practice ^42– 44^. A range of frameworks have been used to guide implementation science research, including the theoretical domains framework (TDF) ^45^, the integrated-Promoting Action on Research Implementation in Health Services (i-PARIHS) ^46^ and the reach, efficacy, adoption, implementation, and maintenance (RE-AIM) framework ^47^. One of the most popular frameworks is the consolidated framework for implementation research (CFIR) ^48^, a determinants framework comprised of 39 constructs under five domains: innovation (e.g., trialability, cost), outer setting (e.g., local conditions, external pressures), inner setting (e.g., culture, available resources), individuals (e.g., mid or high-level leaders, innovation recipients) and implementation process (e.g., engaging, adapting) ^48^. Utilising implementation science may enhance our understanding of determinants to shared models of care for young people with mental health difficulties to inform the delivery of care in Ireland and internationally.

### Rationale & review aims

Previous reviews have synthesised the literature regarding youth mental health ^49^, shared models of care ^50– 52^, and addressing physical health or sexual health needs among populations with mental illness ^53– 57^. Two recent reviews synthesised effective intervention components to integrating youth mental healthcare in community settings ^35, 36^, with a meta-analysis identifying shared models of care were associated with a small but significant improvement in youth’s depression symptoms ^35^. However, to our knowledge, there has yet to be a review of these shared models of care to address the physical and sexual healthcare needs for young people with mental health difficulties. A search of Open Science Framework (OSF), Joanna Briggs Institute (JBI) and Cochrane registries on the 7 ^th^ October, 2024 did not identify any current or completed reviews on this topic. When sexual wellbeing has been included in previous reviews, it has often been consolidated under general physical health concerns and has not been synthesised independently ^35, 36^. Our proposed scoping review aims to bring an implementation science lens, through the use of CFIR, to synthesise the body of literature to identify and describe 1) shared models of care which address the physical health and/or sexual wellbeing of young people with mental health difficulties, and 2) the determinants to implementing these models.

## Protocol

### Methods

This scoping review is the foundation step for a mixed methods study aimed at implementing Shared Care fOr Physical and sExual health (SCOPE for HEALTH) in young people with mental health difficulties in Ireland. This work is part of the broader research programme, VISTA (Vision To Action for Promoting Mental Health and Recovery – An Implementation Science Approach to “Sharing the Vision”), designed to use novel methods to develop implementation blueprints to action a range of recommendations from ‘Sharing the Vision’, Ireland’s national mental health policy ^58, 59^. We are seeking to conduct a scoping review to summarise existing relevant literature on shared models of care as this evidence synthesis approach is well suited to our aims and as we anticipate a high degree of heterogeneity among the included evidence sources ^60^. Review methods have been informed by JBI and protocol reporting has been guided by the Preferred Reporting Items for Systematic Reviews and Meta-Analysis extension for Scoping Reviews (PRISMA-ScR) standards ^61, 62^. The protocol was registered prospectively with OSF on 16 ^th^ October, 2024 ( https://osf.io/xyq9c). Research ethical approval is not required to conduct this review.

### Eligibility criteria

Eligibility criteria is summarised in Table 1. The typical population, intervention, comparator, outcome (PICO) framework is not well suited to this review as we will not have parameters regarding study interventions or outcomes; therefore, we selected the population, concept, context, and type of evidence source framework provided by JBI as it aligns with our review objectives ^61^.

**Table 1.** Summary of eligibility criteria.

	Include	Exclude
**Population**	*•* Young people (ages 10 up to and including 25 years) with mental health difficulties (e.g., symptoms and/or diagnosed concerns) *•* Mental health difficulties as defined by ICD-11	*•* Adults >26 years *•* Animal studies *•* Young people without mental health difficulties *•* Young people with substance use/abuse issues *•* Young people with Autism spectrum or other neurodevelopmental disorders without a mental health comorbidity
**Concept**	*•* Studies which focus on mental health difficulties as the primary concern, and secondary effects on physical health and/or sexual wellbeing *•* Any physical health concern(s) *•* Any sexual wellbeing concern(s) *•* Can address either physical and/or sexual health concerns	*•* Studies focused solely on other aspects of young people’s health (e.g., cognitive, emotional, or social development without explicit links to physical or sexual health) *•* Studies with a primary focus on young people’s physical health and/or sexual wellbeing, with mental health as a secondary concern
**Context**	*•* Shared models of care between primary care and secondary care	*•* No limitations on setting(s)
**Types of** **evidence** **sources**	*•* Quantitative *•* Qualitative *•* Mixed methods *•* Theses *•* Conference abstracts *•* Grey literature	*•* Epidemiological studies *•* Reviews *•* Protocols *•* Text and opinion *•* Book chapters

Studies should include young people, defined as those aged ten and up to, and including, 25 years. This aligns with the definition used to inform ‘Sharing the Vision,’ Ireland’s national mental health policy ^11^. Studies which include young adult and adult populations will be considered for inclusion should the younger adult findings be presented separately from adult data, or when at least 60% of the sample is comprised of the eligible age group ^63^. Studies focused on adults, older adults or animal studies will be excluded. Studies including healthcare providers involved in the provision of mental health, physical health and/or sexual wellbeing care (e.g., general practitioners, psychiatrists, psychologists, practice nurses) will be eligible for inclusion.

We use the term ‘mental health difficulty’ throughout our review to encompass “ *the full range of mental health difficulties that might be encountered, from the psychological distress experienced by many people, to severe mental disorders that affect a smaller population*" as described in ‘Sharing the Vision.’ ^11^ This terminology was also preferred by the public and patient representatives on the review team. Mental or behavioural difficulties included in the International Classification of Diseases- 11 ^th^ Revision (ICD-11) which affect young people will be eligible for inclusion ^64^. Neurodevelopmental conditions as classified by the ICD-11 (e.g., intellectual disabilities, autism spectrum disorder) will be included if they are coupled with a mental or behavioural difficulty. Studies focused solely on substance use or abuse will also be excluded. There will be no limits on the types of physical health or sexual wellbeing concerns identified in the literature.

Studies describing shared models of care between primary and secondary care will be included in the review. Studies will be eligible for inclusion if mental health concerns are the priority focus and physical health and/or sexual wellbeing are secondary concerns. Studies which solely address the effects physical health and sexual wellbeing on mental health will be excluded. There will be no limitations to the types of settings the shared models of care are offered (e.g., sexual health clinics, community or in-patient settings).

The review will consider all published and unpublished literature which address our review objectives. We will consider all study designs, including quantitative, qualitative, and mixed methods designs. Relevant theses will be eligible for inclusion, while conference abstracts, editorials, commentaries and other evidence syntheses will be excluded; however, the reference lists of any related reviews will be hand searched to identify any additional studies to include in our review. Grey literature will be considered where appropriate, and we will contact experts in the field to identify any unpublished research.

### Search strategy & information sources

A comprehensive approach has been used to develop the review search strategy. We initially compiled a list of relevant words associated with the concepts included in the review (i.e., young people, mental health, physical health, sexual wellbeing, models of care and implementation science). The study librarian selected the most relevant terms to include in the development of preliminary search strings in Web of Science (Core Collection) electronic database on 21 ^st^ October, 2024 ( https://osf.io/xyq9c). This initial search will undergo database-specific modifications and be applied to MEDLINE (EBSCOhost), Embase (Elsevier), PsycINFO (EBSCOhost), and CINAHL (EBSCOhost) databases. No geographical, language or time limitations will be placed on the search to ensure all potentially relevant studies are included in the review. Finally, the reference lists of included studies will be hand searched by a team member to identify any additional literature to include in the review. Any non-English literature identified will be translated using DeepL Translate, an online translation aid. All search findings will be imported into Covidence, an online review management platform, to undergo de-duplication and screening (Veritas Health Innovation, Melbourne, Australia).

Grey literature searching will be conducted to complement the electronic database searches. Targeted searches of national health organisation websites and think tanks will be searched by a team member to identify reports, white papers and/or guidance documents which meet our review eligibility criteria. Sites from Australia (e.g., Orygen Institute), Canada (e.g., The Centre for Addictions and Mental Health), the United States (e.g., The Gates Institute for Population and Reproductive Health), the United Kingdom (e.g., The Prince’s Trust), and Scandinavian countries (e.g., Danish Health Authority) will be prioritised as these countries and regions are active in this area of applied research ^65^. Grey literature findings will also be imported into Covidence for further review and data extraction.

### Screening procedures

Title and full-text screening will be conducted by two independent reviewers. Relevant titles and abstracts will have their corresponding full texts imported into Covidence to undergo further review. Full texts will be reviewed again against the eligibility criteria by two reviewers, with reasons for exclusion at this stage noted for the PRISMA flow diagram. Conflicts at each of these stages will be addressed through discussion or with a third team member. Included articles will undergo data extraction.

### Data extraction

A modified version of the JBI extraction template will be used to extract relevant data from included studies to address our review objectives ( https://osf.io/tcj2s). Extracted items will include descriptive study details (e.g., authors and year of publication, country, study design, aim), population details (e.g., characteristics of young people, mental illness, sample size) and concept (e.g., physical health and/or sexual wellbeing concerns), context (e.g., description and setting of shared model of care). Content related to implementation as described by CFIR (e.g., inner setting, outer setting, intervention characteristics, characteristics of the individual, and process) will be extracted when possible. The template will be pilot tested prior to extraction by two of the reviewers using three included studies. Reviewers will agree on the extraction process and resolve discrepancies and any issues with the template. Any revisions made to the template will be described in the final review manuscript. The reviewers will use the updated template to complete data extraction for the remaining included studies. If any essential content is not reported in the study, the research team will email the corresponding author(s) of these studies for supplemental information.

### Critical appraisal of individual sources of evidence

Included studies will undergo critical appraisal using the mixed methods appraisal tool (MMAT) ^66^. The MMAT is a validated tool which allows for the methodological appraisal of quantitative, qualitative and mixed methods studies, and was selected as we anticipate a high level of heterogeneity across included studies ^66^. Each MMAT criterion response is coded as ‘yes’, ‘no’ or ‘can’t tell’ ^66^. Two reviewers will pilot test MMAT coding with three of the included studies and compare results. Any discrepancies will be resolved through consensus discussions. Following pilot testing, the reviewers will continue to complete appraisals independently, with conflicts resolved regularly through consensus discussions. All studies which undergo appraisals will be included in the review, regardless of their methodological quality rating.

### Data synthesis & presentation

 To address our first review objective, we will conduct reflexive thematic analysis ^67^. Following an initial review of the included studies and extracted data, two team members will derive key codes and subcodes in the data which reflect critical concepts of shared models of care and their implementation. Developed codes and subcodes will be categorised into an overarching thematic framework. Extracted study data will then be indexed to the framework and relevant categories. To address the second review objective, we will deductively map identified implementation determinants to relevant domains from CFIR ^48^.

Review findings will be presented according to the PRISMA-ScR reporting checklist ^62^. Data will be aggregated and summarised narratively to address the review objectives, with figures and tables used to support narratives, when appropriate. MMAT appraisals will be presented using a table of aggregated criterion ratings with a supporting narrative. A PRISMA flow diagram will also be included in the findings manuscript to synthesise the review screening process ^68^.

## Discussion

Strengths of this review include using rigorous JBI methods and PRISMA reporting standards for the protocol and finding manuscripts. Registering and publishing the protocol a priori reduces the risk of research duplication and bias, while increasing transparency and replication of our review methods ^69^. Placing no geographical, language or time limiters on the search strategy also helps ensure we identify all relevant literature and will allow us to track changes in characteristics to shared models of care over time. Conducting the optional critical appraisal step in our scoping review will also add rigour to our findings. Limitations may include introducing bias by combining evidence sources, including studies regardless of their quality, and not identifying all relevant sources to include in our review.

Findings from this review will improve our understanding of shared models of care, and barriers, enablers and determinants to implementing these models to support a holistic approach to improving health outcomes among young people. Identifying key characteristics of established shared models of care to address mental, physical health and/or sexual wellbeing needs between primary and secondary care will provide elements to consider replicating in future studies. Mapping review findings to CFIR will highlight key factors influencing implementation and outcomes and uncover potential gaps in the literature. Findings and identified gap(s) in the literature will be used to inform upcoming quantitative and qualitative data collection in our broader mixed methods research study. Finally, findings will inform the tailoring of implementation strategies and health policies for vulnerable populations, thereby supporting more effective delivery of healthcare.

## Ethics and consent

Ethics and consent were not required for this study.

## Abbreviations

CFIR: Consolidated Framework for Implementation Research

ICD-11: International Classification of Diseases- 11 ^th^ Revision

i-PAHRIS: integrated-Promoting Action on Research Implementation in Health Services

JBI: Joanna Briggs Institute

MMAT: Mixed Methods Appraisal Tool

OSF: Open Science Framework

PRISMA: Preferred Reporting Items for Systematic Reviews and Meta-Analyses

RE-AIM: Reach, Efficacy, Adoption, Implementation, and Maintenance

SCOPE for HEALTH: Shared Care fOr Physical and sExual HEALTH in young people with mental health difficulties

ScR: Scoping Review

STI: Sexually Transmitted Infection

WHO: World Health Organisation

## Data Availability

No data are associated with this article. Open Science Framework: Scope for Health PRISMA ScR Checklist. https://osf.io/hwnua/ ^70^. This project contains the following extended data: draft data extraction.docx Data are available under the terms of the Creative Commons Zero "No rights reserved" data waiver (CC0 1.0 Public domain dedication). Open Science Framework: PRISMA-ScR checklist for “Mapping barriers, enablers and implementation determinants to shared models of care for physical health and sexual wellbeing among young people with mental health difficulties using the consolidated framework for implementation research: A scoping review protocol”. https://osf.io/hwnua/ ^70^. Data are available under the terms of the Creative Commons Zero "No rights reserved" data waiver (CC0 1.0 Public domain dedication).
