# Supplementary material for: Mapping barriers, enablers and implementation determinants to shared models of care for physical health and sexual wellbeing among young people with mental health difficulties using the Consolidated Framework for Implementation Research: A scoping review protocol
Source: HRB Open Res. 2025 May 12;8:28. Originally published 2025 Feb 7. [Version 2] doi: 10.12688/hrbopenres.14032.2 (PMC12141993; doi:10.12688/hrbopenres.14032.2)
Supplement: Peer review history

# Reviewer report: Reviewer response for version 2

**DOI:** 10.21956/hrbopenres.15560.r47635

**Published:** 2025-07-21

**Authors:** Melissa Kang

**Affiliations:** 1 The University of Sydney, Sydney, Australia

Thank you for inviting me to review this manuscript, a protocol for a scoping review. The topic and objectives are important to advance the field of health services research in adolescent and youth health. I note that the context of this review relates to/ intends to align with, national mental health policy in Ireland.

 I note that this is a revised manuscript that has been reviewed by (I assume) the two original reviewers. Therefore I provide this brief review to offer any additional questions or comments as the review process and revisions are thorough, detailed and thoughtful.

Terminology and Definitions

 - There is clarity about the definition of young people and justification for the age range in the eligibility criteria, which is important.

 - Can I suggest changing ‘Sexual Health’ to ‘Sexual and Reproductive Health’ ? There is (finally!) increasing use of this expression which is more comprehensive.

 - ‘Mental health difficulty’ – I note the clarity/ definition in using this term in the Eligibility criteria.

Conceptualising holistic care

 While this scoping review aims to consider the physical and sexual (and reproductive) health needs for young people with a mental health difficulty, the ‘starting place’ / ‘index health issue’ is 'mental health difficulty'. This makes sense given that the purpose of the review springs from mental health policy. I argue that a biopsychosocial approach, and integrated care, doesn’t have a starting place that is an ICD-11 or any other diagnostic category but starts with the young person. A chronic or life-threatening physical health condition could lead to mental health difficulties, as could unplanned pregnancy or sexual violence or being in a sexual or gender minority. For the purposes of defining parameters for the scoping review, it is reasonable to start with the population as described in Table 1, but I would like to see this issue of health issue-centred care rather than person-centred care mentioned in the Discussion/ Limitations. Exclusion of neurodevelopmental conditions and substance use  or abuse should also be considered among Limitations since they are generally intertwined with mental health difficulties.

Scope of sexual (and reproductive) health issues

 May I suggest that sexual function be included/ considered – the authors list fertility management, STIs and sexual violence as potential issues; and report on the association between ‘high-risk sexual behaviours’ and mental health difficulties among young people. However there are potential sexual function side effects from commonly prescribed medications (such as SSRIs) and of course strong associations between mental health difficulties (including suicide) among young people from sexuality and/or gender diverse populations.

Mental Health Policy - Sharing the Vision

 It would be interesting to include a brief paragraph which summarises the policy objectives pertaining to young people.

 Please note these are minor comments and I think the manuscript can be published in its current form (with the exception to add ‘reproductive’ in ‘sexual health’.

Is the study design appropriate for the research question?

Yes

Is the rationale for, and objectives of, the study clearly described?

Yes

Are sufficient details of the methods provided to allow replication by others?

Yes

Are the datasets clearly presented in a useable and accessible format?

Yes

Reviewer Expertise:

Health services research and models of care for young people; knowledge translation/ implementation of policy and programs

I confirm that I have read this submission and believe that I have an appropriate level of expertise to confirm that it is of an acceptable scientific standard.

# Reviewer report: Reviewer response for version 2

**DOI:** 10.21956/hrbopenres.15560.r47235

**Published:** 2025-06-05

**Authors:** Anna E Kågesten

**Affiliations:** 1 Karolinska Institutet, Stockholm, Sweden

Thank you to the authors for sharing their revised version. All my comments have been addressed and I have no futther comments to make.

Is the study design appropriate for the research question?

Partly

Is the rationale for, and objectives of, the study clearly described?

Yes

Are sufficient details of the methods provided to allow replication by others?

Yes

Are the datasets clearly presented in a useable and accessible format?

Not applicable

Reviewer Expertise:

Sexual and reproductive health and rights; Sexual wellbeing; Adolescent Health; Systematic and scoping reviews

I confirm that I have read this submission and believe that I have an appropriate level of expertise to confirm that it is of an acceptable scientific standard.

# Reviewer report: Reviewer response for version 2

**DOI:** 10.21956/hrbopenres.15560.r47234

**Published:** 2025-05-28

**Authors:** Ellie Brown

**Affiliations:** 1 The University of Melbourne, Melbourne, Victoria, Australia

Thanks to the reviewers for making the suggested amendments.

Is the study design appropriate for the research question?

Yes

Is the rationale for, and objectives of, the study clearly described?

Yes

Are sufficient details of the methods provided to allow replication by others?

Yes

Are the datasets clearly presented in a useable and accessible format?

Not applicable

Reviewer Expertise:

I am an academic with expertise in youth mental health, models of care, physical health and sexual wellbeing in youth mental health

I confirm that I have read this submission and believe that I have an appropriate level of expertise to confirm that it is of an acceptable scientific standard.

# Reviewer report: Reviewer response for version 1

**DOI:** 10.21956/hrbopenres.15403.r46252

**Published:** 2025-03-26

**Authors:** Ellie Brown

**Affiliations:** 1 The University of Melbourne, Melbourne, Victoria, Australia

Thank you for inviting me to review this manuscript. Some specific comments that I feel would help strengthen it further follow:

 Background - you suggest that severe mental health difficulties are vulnerable to several physical conditions due to antipsychotic medication, but just attributing it to this misses the impact of lifestyle factors as well such as sedatory behaviors and increased smoking and poor diet. I suggest expanding these examples.

 In your eligibility criteria you state 'Studies should include young people, defined as those aged ten and up to, and including, 25 years' but I wonder how you might consider samples that cover up to 30 years old but with a mean age of under 25?

 Discussion: "Findings from this review will improve our understanding of shared model of care" type in model(s)

 Overall this is an important topic, a well written protocol, and I look forward to reading the findings of your scoping review!

Is the study design appropriate for the research question?

Yes

Is the rationale for, and objectives of, the study clearly described?

Yes

Are sufficient details of the methods provided to allow replication by others?

Yes

Are the datasets clearly presented in a useable and accessible format?

Not applicable

Reviewer Expertise:

I am an academic with expertise in youth mental health, models of care, physical health and sexual wellbeing in youth mental health

I confirm that I have read this submission and believe that I have an appropriate level of expertise to confirm that it is of an acceptable scientific standard, however I have significant reservations, as outlined above.

# Response

**Published:** 2025-05-02

**Authors:** Allyson Gallant

**Affiliations:** Public Health & Primary Care, Trinity College Dublin School of Medicine, Dublin, Ireland

1.) We have now added that physical health can be affected by both modifiable individual behaviours and mental health treatment plans. Please see page 4 for this update and additional reference

 2.) We spent a long time within our group considering and discussing the age range of interest. Ultimately, we landed on utilising the age range within Ireland’s national mental health policy as our guide. The ‘bigger picture’ purpose of this review (alongside other activities) will be to inform the design of a new model of shared care for youth with mental health difficulties as regards to their physical and sexual wellbeing. We are therefore cognisant to keep our framework within the scope of the relevant national policies and the broader health services that will be tasked with implementation of any future model.  We have added a few additional lines to the initial Methods paragraph to address this (please see Page 8). 

 3.) Thank you for these thoughtful comments and catching the typo! We have now addressed this on page 13.

# Reviewer report: Reviewer response for version 1

**DOI:** 10.21956/hrbopenres.15403.r45551

**Published:** 2025-03-24

**Authors:** Anna E Kågesten

**Affiliations:** 1 Karolinska Institutet, Stockholm, Sweden

Thank you for the opportunity to review this paper, which I have read with great interest. This is an overall well-written protocol for a scoping review mapping barriers, enablers and implementation enablers for shared models of care in relation to physical and sexual wellbeing among young people with mental health difficulties. The focus of the review on a specific topic, which has not been extensively mapped before, is a key strength. In addition, the CFIR is suitable given the implementation focus. 

 However, there are a number of places where the protocol would benefit from additional details or clarification. Below I have included some major points for the author's consideration. 

 First, Please clarify the rationale behind choosing a scoping review design for this paper, as it seems more aligned with a systematic review approach. Typically, a scoping review does not involve quality assessments or critical appraisal of included studies, as planned in this case. Instead, it usually aims to map existing evidence on a topic rather than to determine causality or what influences what.

 Considering your second aim, which focuses on determinants of shared care models, it is unclear why a systematic review is not being conducted. Additionally, I am curious about the overall strength of the evidence generated from the review, particularly in relation to understanding “determinants.” If you are using a thematic approach to synthesizing findings, will you be applying tools such as GRADE-CERQual to assess the quality and confidence of the evidence? Clarifying this aspect would strengthen the methodological rigor.

 Second, I am not familiar with the use of reflexive thematic analysis in the context of a scoping review (or even a systematic review), as this method is traditionally applied to qualitative data. In your paper, it seems that you are combining different study designs. It would be helpful to clarify how you plan to differentiate and code quantitative versus qualitative findings.

 Furthermore, the use of thematic synthesis (distinct from thematic analysis) is typically suited for qualitative studies, whereas thematic summary can be applied to quantitative studies. You also mention that the data will be aggregated and summarized narratively, with figures used to support narratives. Could you specify whether this approach is consistent with reflective thematic analysis, and clearly define what you mean by “narratives” in this context? Are these the main themes identified, or are they related to another analytical approach?

 Third, the definition of “sexual health and wellbeing” cited in the introduction is not commonly used or widely recognized within the field of Sexual and Reproductive Health and Rights (SRHR). These two concepts, while interrelated, are distinct and often conceptualized differently.

 To enhance clarity and alignment with existing literature, consider referencing more established definitions or frameworks, such as:

- Mitchell et al.’s framework for sexual wellbeing, which highlights the relationship between health, wellbeing, rights, and justice. [Ref 1]
- The Guttmacher-Lancet integrated definition of SRHR, which comprehensively covers related concepts. [Ref 2]

Additionally, the term “common sexual health concerns” among youth does not accurately reflect the most prevalent issues from a global perspective. I recommend adding unintended pregnancy, HIV/STIs, and sexual violence, rather than issues such as cervical cancer screening, as it is typically not a routine clinical service offered to youth (particularly those under the age of 25). If the issues listed are specific to the local study context, then please clarify this in the text. 

 Please also clarify the definition of "youth" used in the review. Currently, it seems to include individuals aged 25, which is higher than the international standard definitions (e.g., WHO defines “youth” as 15-24 years and “young people” as 10-24 years). Would it be more appropriate to refer to those aged 25-30 as “young adults” instead?

 Finally, the only limitation mentioned in the paper is that not all relevant studies may be identified. While this is a common and expected limitation of any review, it would be helpful to acknowledge other potential limitations specific to your approach. For example: biases introduced by combining qualitative and quantitative data without clear differentiation; and the challenge of assessing evidence quality when using diverse study designs.

Is the study design appropriate for the research question?

Partly

Is the rationale for, and objectives of, the study clearly described?

Yes

Are sufficient details of the methods provided to allow replication by others?

Yes

Are the datasets clearly presented in a useable and accessible format?

Not applicable

Reviewer Expertise:

Sexual and reproductive health and rights; Sexual wellbeing; Adolescent Health; Systematic and scoping reviews

I confirm that I have read this submission and believe that I have an appropriate level of expertise to confirm that it is of an acceptable scientific standard, however I have significant reservations, as outlined above.

## References

[1] : What is sexual wellbeing and why does it matter for public health?. *Lancet Public Health* .2021;6(8) : 10.1016/S2468-2667(21)00099-2 e608-e613 10.1016/S2468-2667(21)00099-2 34166629 PMC7616985

[2] : Accelerate progress-sexual and reproductive health and rights for all: report of the Guttmacher-Lancet Commission. *Lancet* .2018;391(10140) : 10.1016/S0140-6736(18)30293-9 2642-2692 10.1016/S0140-6736(18)30293-9 29753597

# Response

**Published:** 2025-05-02

**Authors:** Allyson Gallant

**Affiliations:** Public Health & Primary Care, Trinity College Dublin School of Medicine, Dublin, Ireland

Thank you for these comments on our protocol and the opportunity to enhance our review.  

 1.) First, we are aiming to conduct a scoping review rather than a systematic review as we are seeking to identify the breadth of evidence on shared models of care to summarise what has previously been done, rather than to answer a specific, in-depth question or identify effective components of shared models of care as with a systematic review.  

 Additionally, we would likely be unable to do an adequate synthesis of the evidence to complete a systematic review as we anticipate a high level of heterogeneity in both the literature sources and their quality. Summarising the breadth of evidence will likely be a more sensible approach to address our aims compared to a systematic review. We have added a few more details on Page 8 and Page 12, and a new citation (highlighted below) to the manuscript to help address this.  

 Finally, the critical appraisals of included sources is an optional step on a scoping review as outlined by JBI and the PRISMA-ScR reporting guidelines. We will also be including all evidence sources regardless of the critical appraisal/MMAT scores, which can differ from typical systematic reviews. Our critical appraisal will therefore inform our understanding of the quality of the existing research, affording us the opportunity to include a commentary on quality rather than utilising quality appraisal as a mechanism for exclusion of studies.   

 Reference: 

 Munn, Z., Peters, M. D., Stern, C., Tufanaru, C., McArthur, A., & Aromataris, E. (2018). Systematic review or scoping review? Guidance for authors when choosing between a systematic or scoping review approach. BMC medical research methodology, 18, 1-7. 

 2.) The use of reflexive thematic analysis has been previously used in other reviews by research team members and in mental health/implementation science systematic reviews (see citations below).  

 When we refer to “narratives” in our proposed approach, we mean the analytically constructed accounts that represent patterns of shared meaning—namely, the main themes generated through iterative analysis. These narratives are not participant case studies or chronological storylines but rather represent the interpretative presentation of the central themes identified through coding and theme development. Any figures included will be intended to illustrate and support these themes, providing visual clarity to aspects of the analysis (e.g., thematic maps, frequency counts of theme prevalence if appropriate, or illustrative quotes), rather than to quantify findings in a positivist sense. 

 References: 

 O’connell, N., O’connor, K., McGrath, D., Vagge, L., Mockler, D., Jennings, R., & Darker, C. D. (2022). Early Intervention in Psychosis services: A systematic review and narrative synthesis of the barriers and facilitators to implementation. European Psychiatry, 65(1), e2. 

 Geerligs, L., Rankin, N. M., Shepherd, H. L., & Butow, P. (2018). Hospital-based interventions: a systematic review of staff-reported barriers and facilitators to implementation processes. Implementation Science, 13, 1-17. 

 3.) Thank you for these insights and additional references to consider. We have revised the definition we used for sexual health & wellbeing to a more suitable international context, using the Mitchell et al (2021) framework suggested. Please see page 5 for the revised terminology.  

 4.)We have revised this section in the manuscript to better reflect the current health needs and concerns of youth, based on a new WHO report. Please see revisions on Page 5. 

 5.) We spent a long time within our group considering and discussing the age range of interest. Ultimately, we landed on utilising the age range within Ireland’s national mental health policy as our guide. The ‘bigger picture’ purpose of this review (alongside other activities) will be to inform the design of a new model of shared care for youth with mental health difficulties as regards to their physical and sexual wellbeing. We are therefore cognisant to keep our framework within the scope of the relevant national policies and the broader health services that will be tasked with implementation of any future model.  We have added a few additional lines to the initial Methods paragraph to address this (please see Page 8).

 6.) We have now revised this paragraph to be more comprehensive. Please see page 12.
